# Call to action: improving primary care for women with COPD

**DOI:** 10.1038/s41533-017-0013-2

**Published:** 2017-02-15

**Authors:** Ioanna Tsiligianni, Miguel Román Rodríguez, Karin Lisspers, Tze LeeTan, Antonio Infantino

**Affiliations:** 1 Clinic of Social and Family Medicine, Faculty of Medicine, University of Crete, Heraklion, Crete Greece; 2 Son Pisà Primary Care Health Center, Majorca, Baleares Spain; 3 Instituto de Investigación Sanitaria de Mallorca (IdISPa) Baleares, Majorca, Spain; 4 Department of Public Health and Caring Sciences, Family Medicine and Preventive Medicine, Uppsala University, Uppsala, Sweden; 5 The Edinburgh Clinic 306 Choa Chu Kang Ave 4 #01-685, Singapore, 680306 Singapore; 6 Italian Interdisciplinary Society for Primary Care, Casamassima Bari, Italy

## Abstract

In this perspective-based article, which is based on findings from a comprehensive literature search, we discuss the significant and growing burden of chronic obstructive pulmonary disease in women worldwide. Chronic obstructive pulmonary disease now affects both men and women almost equally. Despite this, there remains an outdated perception of chronic obstructive pulmonary disease as a male-dominated disease. Primary care physicians play a central role in overseeing the multidisciplinary care of women with chronic obstructive pulmonary disease. Many women with chronic obstructive pulmonary disease delay seeking medical assistance, due to fear of stigmatization or dismissing symptoms as a ‘smoker’s cough’. Improving awareness is important to encourage women with symptoms to seek advice earlier. Once women do seek help, primary care physicians need to have knowledge of the nuances of female chronic obstructive pulmonary disease disease presentation to avoid mis- or delayed diagnosis, both of which are more common in women with chronic obstructive pulmonary disease than men. Subsequent management should consider gender-specific issues, such as differential incidences of comorbid conditions, potentially higher symptom burden, and a higher risk of exacerbations. Chronic obstructive pulmonary disease treatment and smoking cessation management should be specifically tailored to the individual woman and reviewed regularly to optimize patient outcomes. Finally, education should be an integral part of managing chronic obstructive pulmonary disease in women as it will help to empower them to take control of their disease.

## Introduction

The burden of chronic obstructive pulmonary disease (COPD) in women is considerable and growing. In fact, COPD in women is now responsible for more female deaths than many of the most commonly recognized forms of cancer.^1^ Despite the enormity of this burden, there is often a disregard for COPD as a women’s healthcare issue. One of the underlying reasons for this may be the inaccurate, outdated perception that COPD is a disease of older, male-smokers, combined with a lack of awareness of symptoms amongst women. In addition, women are often embarrassed by symptoms such as cough and the stigma associated with it (particularly in current ex-smokers), which can result in delayed diagnosis.

COPD symptoms are debilitating, frightening, and sometimes life-threatening. Patients report feelings of guilt, shame, and isolation and may be unable to fulfill their established roles within the family and workplace. Much can be done to improve the lives of these patients, but a greater awareness is required of the gender-specific disease characteristics and issues.

The primary care physician plays a central role in the multidisciplinary team involved in the diagnosis and treatment of women with COPD. In this perspectives piece, we provide a ‘call to action’ for those working in primary care to advocate for better awareness, prevention and gender-focused management for the millions of women with COPD worldwide. To gather the evidence to support this call we conducted a review based on PubMed searches using the terms: women, woman, female, gender, COPD, and primary care. Relevant cross-referenced articles, including Cochrane evidence, and COPD guidelines were reviewed. The initial search retrieved over 3000 articles, from which 177 were selected based on direct comparisons between male and female groups or specific reference to gender-related issues. In this paper we have included those we considered most pertinent to the focused topics we discuss.

## The increasing burden of COPD in women

The prevalence of COPD in women varies by country; however, most evidence indicates similar disease prevalence in men and women.^2–7^ A structured review of studies estimating the epidemiology of COPD included data from Canada, Italy, Sweden, the UK, and the USA; a mostly similar prevalence in men and women was reported, with estimates of prevalence in women ranging from ~3% to ~15%.^4^ These prevalence estimates are broadly similar to those reported in other studies and challenge the outdated perception of COPD as a disease that predominantly impacts men.^8^

Furthermore, the prevalence of COPD in women is continuing to rise. In a Dutch study, COPD prevalence in women increased from 37.5/1,000 patients in 1980 to 47.2/1,000 patients in 2006, whereas a marked decline was observed for men over the same time period (from 115.2/1,000 patients to 59.0/1,000 patients).^2^ In England, the lifetime prevalence of COPD in women increased from 12.6/1,000 patients in 2001 to 16.2/1,000 patients in 2005.^9^

A number of factors are likely to have contributed to the increasing prevalence of COPD in women, including increases in smoking prevalence in some countries and exposure to other risk factors (such as the use of biomass smoke for cooking and heating) and occupational risk factors such as fish smoking).^5^

Smoking prevalence varies widely by country, region, and sociodemographic status. In some high-income countries, there have been declines over recent years in the incidence of tobacco smoking; however, a 15–20-year lag time between smoking and COPD onset means that, even in these countries, the burden of decades of increasing tobacco use by women is likely to be reflected in increasing COPD prevalence for some time to come.^4, 10–16^ In the United States between 1999 and 2009, total COPD-related deaths increased by 19% in women but only 5% in men.^17^ This increase is likely to reflect the decade delay in peak smoking prevalence in women vs. men.^12^

In populations in which biomass fuel is used (most frequently in low-income countries), women often bear a disproportionate burden of exposure to biomass smoke, due to a greater role in cooking and domestic responsibilities. In the FRESH-AIR Uganda study, a COPD prevalence of 16.8% was reported among women with a mean age of 45.4 years, with the highest prevalence in those aged 30–39 years.^18^ Most (74%) were never smokers but almost all (92.0%) had been exposed to smoke from indoor and/or outdoor biomass fuel for most of their lives. Likewise, European countries that have faced financial crisis have seen an increase in indoor smoke pollution due to wood burning for heating culminating in a deterioration of symptoms in people with COPD.^19^

COPD mortality data can also be used as an indicator of disease burden. In Sweden, COPD mortality in women increased between 1999–2009, and life expectancy among COPD patients was 9.4 years lower in women (vs. 7.4 years lower in men) compared with the average Swedish population.^20^ Furthermore, surveillance data (1971–2000) from the United States showed in 2000, for the first time, that the number of COPD deaths recorded in women surpassed the number of COPD deaths among men.^21^

## Improving diagnosis in women with COPD

Under-diagnosis or mis-diagnosis of COPD occurs more frequently in women than men, this may be partly attributable to a gender bias that exists in the outdated perception of COPD as a male-dominated disease.^1, 13, 22–26^ In the Epidemiologic Study of COPD in Spain (EPI-SCAN), the odds ratio for being correctly diagnosed with COPD was 1.9 for men vs. women.^13^ In addition, many women are unaware of the key symptoms of COPD, resulting in a failure to report their symptoms to their primary care physician.^27^ As a result of these combined factors, diagnosis is often delayed in women, meaning that COPD may be well advanced before treatment starts.

Primary care consultations should include a careful obtainment of patient history, including the use of symptom-based questionnaires, followed by a thorough physical examination.^28–30^ The use of validated questionnaires can be useful to facilitate case finding and monitoring.^30, 31^ A diagnosis of COPD should be considered in any woman with prior or current risk factor exposure, such as respiratory infection (Table 1), presenting with symptoms such as breathlessness, chronic cough, regular sputum production or wheeze, and/or with a positive result on a COPD risk evaluation questionnaire.^30^ Physicians should be alert to the early identification and diagnosis of COPD in women due to the increased use of tobacco among women in high-income countries, and because of a higher risk of exposure to indoor air pollutants (e.g. biomass smoke from cooking and heating) in women from low-income countries.^5, 32^ Physicians should also be aware that, compared with men, women presenting with COPD may be younger, smoke less and have a lower socioeconomic status.^1, 33–35^ A number of studies have indicated that women may be more susceptible to lung damage than men for the same level of risk exposure, possibly due to lung size and the influence of sex hormones.^36, 37^ Primary care physicians should be alert to these differences to facilitate early recognition of COPD in women. Moreover, to reduce the potential for misdiagnosis of asthma in women, physicians should bear in mind that women with symptoms of COPD are more likely to be diagnosed with asthma than men with symptoms of COPD.^22^

**Table 1:** Known risk factors for COPD in women^29^

Risk factor
• Tobacco smoke exposure (primary and secondary)
• Exposure to biomass fuel smoke
• Occupational exposure
• Low socioeconomic status
• Presence of respiratory infection

Full spirometry with bronchodilation should be used to confirm a COPD diagnosis and lead to the initiation of treatment.^22, 29^ Alternatively, micro-spirometers may enable primary healthcare professionals to exclude patients at risk but with normal lung function values from undergoing a full spirometry, and to identify patients who require further investigation with spirometry and/or referral to a pulmonary specialist.^30^ Research has indicated that spirometry may be underused in women.^22^

**Author suggestions for improving the diagnosis of COPD in women**
- Primary care physicians should be aware of the COPD diagnosis bias in favor of men and that women are often misdiagnosed with asthma.
- Proper processes should be followed, including the use of validated questionnaires and micro-spirometry to facilitate early diagnosis and monitoring.
- Spirometry is mandatory to confirm a COPD diagnosis but it is underused in women, so increased awareness and utilization is suggested.
- Familiarity with gender-specific characteristics will facilitate prompt COPD diagnosis (e.g. younger age, lower BMI, greater risk for lung impairment and severe dyspnea for the same level of smoke exposure, and lower socioeconomic status than men).
- COPD diagnosis should be considered in women with prior or current risk factor exposure (not only active smoking, but also passive smoking and indoor smoking from cooking, heating, etc.), presenting with symptoms such as breathlessness, chronic cough, regular sputum production or wheeze, and in later stages, dyspnea.

## Barriers to accessing healthcare for women with COPD

Many women with COPD dismiss their symptoms as ‘smoker’s cough’ and do not seek medical help.^3^ Using validated screening questionnaires could help identify COPD in such patients.^3, 30, 31^ In addition, the coughing associated with COPD is associated with a social stigma (particularly in current, or ex-smokers) that may result in feelings of embarrassment, guilt and shame among women, making them reluctant to seek care.^38^ Poverty is more common in women than men, particularly older women, and may impact on access to healthcare and obtaining a timely diagnosis.^38, 39^ When women do approach their healthcare provider with respiratory symptoms, they may not receive appropriate intervention because physicians are less likely to recognize COPD in women than men.^13, 25, 26^

**Author suggestions to limit healthcare access barriers for women with COPD**
- Community initiatives help to increase COPD awareness in women and reduce the stigma associated with the disease, which may be a barrier to accessing healthcare. These campaigns need to be primary care oriented and sustained to remain impactful.
- Social support programmes should be implemented to ensure women at risk of COPD of low socioeconomic status can access healthcare.

## Achieving goals in the treatment of women with COPD

The primary goal of treatment in women with COPD is to reduce the disease burden and prevent or slow disease progression. To achieve this, careful consideration should be given to the physical limitations, quality of life and psychological problems, as well as gender-specific factors that may require modification of treatment.^29, 40^ In the overall COPD population, breathlessness is the most commonly reported symptom, followed by fatigue, but women may experience more problems with shortness of breath than men.^29, 40^ COPD exacerbations occur more frequently in women than men,^41, 42^ so monitoring exacerbation risk and implementing appropriate management plans are particularly important in women with COPD. Studies suggest that women with COPD experience a more impaired quality of life at an earlier age of onset than men.^43–46^ Some comorbidities, e.g. psychological issues, are more common in women than men with COPD and are also associated with poorer quality of life.^40^

Most interventions to achieve goals of treatment in women with COPD can be conducted in primary care. Table 2 highlights some particular considerations when treating women with COPD; for example, it is known that women may find it more challenging to stop smoking vs men. Indeed, some evidence suggests that cessation success is less than half as likely in women compared with men after 12 months, indicating the need for different approaches between genders.^47, 48^ Educating women about their COPD, including risk avoidance, early recognition of symptoms and the need to seek medical help, physical exercise, diet, and self-management, empowers women to take control of their disease (Table 3). Primary care physicians should seek and coordinate multidisciplinary input for ensuring optimal COPD management.^29^

**Author suggestions for improving the treatment of COPD in women**
- Does the patient understand the importance of lifestyle interventions, such as smoking cessation and physical exercise? Emphasize the additional benefits of smoking cessation in women compared with men and address issues that may be important to women, such as weight gain concerns. Remember to advise pregnant women about the risks of smoking/second-hand smoke and take action.
- Is the prescribed inhaler the most appropriate to meet the patient’s needs? Consider the potentially higher symptom burden in women compared with men.
- Be aware of the increased exacerbation risk in women vs. men and consistently monitor potential indicators, e.g. sputum production and antibiotic use. Make women aware of the signs of exacerbations and how to handle them.
- Have you considered your patient’s quality of life? Bear in mind that quality of life in women with COPD can be impaired at an earlier stage of disease than in men.

**Table 2:** Considerations for COPD interventions in women

Intervention	Special considerations in women
Smoking cessation	• Women often find it more difficult to quit smoking than men and may need a different behavioral and pharmacological approach^47^
Vaccinations	• As appropriate, for the prevention/treatment of exacerbations (e.g. influenza vaccine)
Pharmacological treatments	• National and international guidelines should be followed
	• Care should be taken to ensure that the treatment administered does not worsen common comorbidities, and to be aware that some medications used to treat comorbidities may have a beneficial effect in COPD as well
Pulmonary rehabilitation	• Focus on common symptoms in women, e.g. breathlessness, anxiety/depression
Oxygen therapy	• As appropriate, for chronic respiratory failure

**Table 3:** Educating women about their COPD for better disease control

Topic	Special considerations in women
1. Risk avoidance, e.g. smoking cessation and avoidance of passive smoke exposure	Women may find smoking cessation more challenging than men so this should be considered in the level of intervention and advice provided.^54^ However, smoking cessation is associated with even greater benefits in women than men,^55^ reinforcing the benefits of investing effort into this intervention
2. Early recognition of symptoms/exacerbations and seeking medical help	Women may dismiss their symptoms as a smoker’s cough^3^ and there may be a tendency for women to feel embarrassed/guilty.^39^ Women are more prone to exacerbations so they should be able to identify signs of exacerbations earlier and be able to react to them. Campaigns to remove social stigma and increase symptom awareness may encourage women to seek medical help earlier
3. Benefits of physical exercise	Tailored specifically for women
4. Dietary advice	To ensure maintenance of a healthy body weight and reducing risk of certain comorbidities (e.g. osteoporosis)
5. Symptom management	The symptom burden in women with COPD may be greater than in men with COPD
6. Inhalation technique	As appropriate
7. Treatment plans	To include exacerbation action plans to encourage women to respond to increased symptoms

## Treating comorbidities in women with COPD

Comorbidities, including cardiovascular disease, lung cancer, gastroesophageal reflux and osteoporosis, for example, are common in patients with COPD, however some, such as asthma, osteoporosis, anxiety, and depression are more common in women than men.^26, 40^ Managing the psychological aspects of COPD is an extremely important part of the overall COPD care package. Women are more likely to experience anxiety and depression, which has detrimental impact on overall quality of life.^40^

Furthermore, for similar levels of lung function decline, women may also be at greater risk of lung cancer than men.^49^ Consistent with previous findings, the recent Majorca Real-Life Investigation in COPD and Asthma cohort study showed that diagnoses of overlapping asthma and COPD were more common (53.4%) in women than men (46.6%).^26^ Primary care physicians are in the best position to recognize the potential impact of comorbidities and ensure they are treated in a timely manner.^29^ In addition, decisions about the choice of pharmacological intervention for COPD should take comorbidities into account. Indeed, it may be that certain medications used to treat comorbidities can offer an additional effect that could be of benefit for patients with COPD. For example, studies in postmenopausal women have shown that those women receiving hormone replacement therapy experience a slowing of pulmonary function decline and reduced inflammation compared with women who do not receive exogenous hormone treatment.^50^ Given the increased prevalence of osteoporosis in women with COPD vs. men, prescribing ICS in women at risk of this disorder warrants careful consideration.^51^ This may be of particular relevance as some evidence suggests higher rates of ICS prescriptions in women vs. men.^52^ Advice regarding the benefits of physical activity should be given, bone mineral density monitored and treatments prescribed as appropriate.

**Author suggestions for improving the treatment of comorbidities in women with COPD**
- Check for evidence of unrecognized/untreated comorbidities, paying particular attention to those that are more common in women and adjust therapy accordingly.
- If a patient is being treated for multiple conditions, consider whether they are coping with the treatment burden? Are they adhering to treatment? Do they understand what each medication is for?
- Are there any COPD or other medications that should be avoided because of specific comorbidities? Alternatively, are there medications available that could have an additional beneficial effect for COPD and comorbidity?

## Patient reviews

Women with COPD should be regularly reviewed to optimize treatment and slow disease progression.^29^ It is helpful to assess disease severity, risk of exacerbations and impact of the disease on the patient at each review, keeping gender-specific issues in mind.^41, 42, 53^ Smoking should be assessed and any concerns addressed in a structured way. The use of COPD-specific quality of life questionnaires (e.g. Clinical COPD Questionnaire [CCQ], COPD Assessment Test [CAT]) and spirometry may be useful at reviews, and any patients who have a rapid decline in lung function or an abnormal symptomatic change may need to be referred to pulmonary specialists.

Primary care physicians should use these sessions as an opportunity to reinforce disease education, tailored to women’s needs. Pharmacological interventions should also be reviewed based on requirement and gender-specific issues, such as ensuring women with COPD receive adequate treatment and considering the potential for increased symptom burden and exacerbation risk in women vs. men with COPD. Appropriate use of oral corticosteroids should also be assessed bearing in mind the high risk for osteoporosis in women with COPD.^51^

**Author suggestions for improving follow-up in women with COPD**
- Use patient reviews as an opportunity to optimize both physician-led and patient-led disease management; be aware of gender-specific factors and routinely assess for the occurrence of female-dominant comorbidities.
- Always use a structured validated questionnaire to assess quality of life as evidence suggests that women with COPD may be particularly vulnerable to impaired quality of life from an early stage of disease (e.g. CCQ-CAT).
- Make women aware of the signs of exacerbations and how to handle them.
- Does your patient understand how to manage their disease and what to do when symptoms deteriorate? Highlight specific issues for women with COPD (for example, more careful use of oral corticosteroids).

## Summary

Millions of women worldwide suffer as a result of COPD. Primary care physicians play a pivotal role in the identification and treatment of women with COPD and must take positive steps to improve the health and well-being of women in their care. Increasing awareness of COPD in women, involving a multidisciplinary team in their treatment and empowering women with COPD through education are vital steps in this process.
